# 肺癌血清肿瘤标志物*meta*分析

**DOI:** 10.3779/j.issn.1009-3419.2010.12.11

**Published:** 2010-12-20

**Authors:** 先锋 卢, 雪琴 杨, 志敏 张, 东 王

**Affiliations:** 400042 重庆，第三军医大学大坪医院野战外科研究所肿瘤中心 Cancer Center, Daping Hospital and Surgery Research Institute, Tird Military Medical University, Chongqing 400042, China

**Keywords:** 肺肿瘤, *meta*分析, 肿瘤标记物, Lung neoplasms, *meta*-analysis, Tumor markers

## Abstract

**背景与目的:**

血清肿瘤标志物的检测对肺癌的早期诊断有着重要的意义。本研究旨在通过回顾性统计分析，总结肺癌血清肿瘤标志物检测的临床意义及特点。

**方法:**

应用*meta*分析方法对有关肺癌血清肿瘤标志物的文献进行综合定量评价。入选712篇文献，病例组、对照组病例均 > 20例，共统计肿瘤血清52 832例，对照血清32 037例。

**结果:**

13项肿瘤标志物对肺癌具有诊断意义，CEA、CA125、CYFRA21-1、TPA、SCCAg、DKK1、NSE和ProGRP的敏感度分别为47.50%、50.11%、57.00%、50.93%、49.00%、69.50%、39.73%和51.48%，特异度分别为92.34%、80.19%、90.16%、88.41%、91.07%、92.20%、89.11%和94.89%。多指标联合分析显示，NSE+ProGRP对小细胞肺癌的诊断敏感度和特异度分别为88.90%和72.82%。SCCAg+CYFRA21-1联合肿瘤生长因子TSGF对鳞癌诊断的敏感度和特异度分别为95.30%和74.20%；CA153+Ferrtin+CEA对肺癌诊断的敏感度和特异度分别为91.90%和44.00%。

**结论:**

肿瘤血清标志物对早期肺癌的诊断有临床意义，单项指标检测敏感度及特异度较低，多指标联合检测能提高诊断的敏感度和特异度。

## Abstract (en)

**Background and objective:**

The detection of serum tumor markers is of great value for early diagnosis of lung cancer. The aim of this study is to summarize the clinic significance characteristics of serum markers contributing to the detection of lung cancer.

**Methods:**

References about serum markers of lung cancer were estimated using *meta*-analysis method. 712 references which included more than 20 cases, 20 controls, the serum markers of 52 832 patients with malignancies and 32 037 patients as controls were evaluated.

**Results:**

Overall the detection of 13 markers play a significant part in lung cancer diagnosis. The sensitivity of CEA, CA125, CYFRA21-1, TPA, SCCAg, DKK1, NSE, ProGRP in the patients' serum with lung cancer were 47.50%, 50.11%, 57.00%, 50.93%, 49.00%, 69.50%, 39.73%, 51.48% and the specificity were 92.34%, 80.19%, 90.16%, 88.41%, 91.07%, 92.20%, 89.11%, 94.89%. In the combined analysis of tumor markers: the sensitivity, specificity of NSE+ProGRP were 88.90% and 72.82% in diagnosis of small cell lung cancer, respectively. In diagnosis of squamous corcinoma, the sensitivity and specificity of TSGF+SCCAg+CYFRA21-1 were 95.30% and 74.20%. The the sensitivity and specificity of CA153+Ferrtin+CEA were 91.90% and 44.00% in diagnosis of lung cancer.

**Conclusion:**

Although the assay of tumor markers in serum is useful for diagnosis of early lung cancer, the sensitivity and specificity are low. Combined detection of these tumor markers could increase sensitivity and specificity.

肺癌作为全球发病率及死亡率第一的恶性肿瘤性疾病，早期诊断和早期治疗是预防肺癌发生和降低死亡率最有效办法。当前广泛运用的检测手段包括无创检查（如X线、CT、钼靶摄片等）和有创检查（纤维支气管镜、胃肠镜活检、支气管造影、B超或CT定位下胸腹腔包块穿刺活检等），因缺乏依从性和普及运用的可能，确诊的肺癌患者80%已属于晚期，5年生存率低于15%^[1]^，而早期诊断的肺癌患者，5年生存率却高达80%，根治术后（包括进展期）5年生存率达35%-50%。肿瘤标志物监测对肺癌的诊断和治疗及预后有重要的临床意义。本文通过*meta*分析系统性回顾1994年1月-2009年9月在Pubmed及中文数据库中发表的关于肺癌血清肿瘤标志物的相关文献，探讨肿瘤标志物在早期肺癌诊断中的临床意义及特点。

## 1 材料和方法

### 1.1 文献纳入标准和排除标准

纳入标准：①国际国内发表的关于肺癌血清肿瘤标志物的文献资料，参考文献语言限定为英文和中文；②因摘要并不能提供完整的相关信息，故只有全文资料被采纳；③各项研究相对独立，考虑到了标本量的大小对实验结果准确性的影响，病例组、良性病变组及对照组病例数均 > 20；④文献资料的研究方法方向相似（都采用了病例-对照方法），且均有完善的数据指标。排除标准：①质量差：数据资料不全、重复报告的文献；②非原发性肺癌，如转移癌、复发癌；③经过临床治疗干预后的血清肿瘤标志物探讨文献。

### 1.2 文献检索

#### 1.2.1 检索数据库

在Pubmed数据库和中国期刊网全文数据库、中文科技期刊全文数据库、万方数据库、中国生物医学文献数据库、中文生物医学期刊目次数据库（CMCC）、中国医学学术会议论文数据库以及手工检索，搜索1994年1月-2009年9月发表的相关文献。

#### 1.2.2 检索策略

关键词：“肺癌”、“*meta*分析”、“小细胞肺癌”、“非小细胞肺癌”、“腺癌”、“鳞状细胞癌”、“检测”、“诊断”、“血浆”、“血清”、“蛋白”、“标记物”和“肿瘤标记物”，通过对初始文献的搜索，以及对更多有关联的文献进行调查，首先纳入题目相关文献，针对其摘要进行筛取。其次，采纳了在Pubmed文献目录和中文数据库的相关索引。

### 1.3 文献资料提取

#### 1.3.1 研究信息的提取

包括作者、发表时间、国家、研究的类型（前瞻性或回顾性）。

#### 1.3.2 研究对象

病例组为临床病理诊断、肺部重复X摄片或CT摄片确诊病例；对照组为健康人群及肺部良性病变组，健康组来源于医院体检人群，良性病变包括肺部炎性病变、肺心病、肺结核等；所有研究对象均不限定民族、年龄、性别。

### 1.4 诊断参数信息的提取

包括敏感度、特异度、诊断准确性，以上诊断参数的定义和计算公式见参考文献^[2]^。

### 1.5 统计学方法

采用Meta-Disc 1.4和SPSS 12.0软件行统计学处理^[3]^。

#### 1.5.1 异质性检验

根据*χ*^2^检验结果，根据相应的自由度行比较，若*P* < 0.05，拒绝同质性假设，说明纳入研究间有异质性，选择随机效应模型进行*meta*分析；反之，*P* > 0.05，说明纳入研究间具有同质性，选择固定效应模型进行*meta*分析。

#### 1.5.2 *meta*分析

按照相应的效应模型，首先将各项研究的敏感度和特异度进行*Logit*变换，再按照权重大小进行总结，最后进行反*Logit*变换得出加权汇总敏感度和特异度。

#### 1.5.3 敏感度分析

将纳入研究逐一排除后，对剩余的文献进行*meta*分析，评价汇总敏感度和特异度。若结果变化大，说明纳入文献的稳定性较好；反之，纳入文献的稳定较差。

## 2 结果

根据以上文献资料的入选及排除标准，经筛选并纳入本次*meta*分析的文献共有712篇，累计病例52 832例，对照32 037例。

### 2.1 单项指标统计结果

共检索出16项血清肿瘤标志物对肺癌诊断有临床价值，按纳入文献标准进行筛选，13项行*meta*分析，在国内文献统计中，腺癌敏感标志物为癌胚抗原（carcino-embryonic antigen, CEA）（敏感度47.50%、特异度92.34%）、糖类抗原125（carbohydrate antigen, CA125）（敏感度50.11%、特异度80.19%）；鳞癌敏感标志物为细胞角蛋白19片段（cytokeratin 19 fragment, CYFRA21-1）（敏感度57.00%、特异度90.16%）、组织多肽特异性抗原（tissue polypeptide antigen, TPA）（敏感度50.93%、特异度88.41%）、鳞状上皮细胞癌抗原（squamous cell carcinoma antigen, SCCAg）（敏感度49.00%、特异度91.07%）；小细胞肺癌敏感标志物为Wnt通路抑制因子Dickkopf-1（DKK-1）（敏感度69.50%、特异度92.20%）、神经元特异性烯醇化酶（neuron-specific enolase, NSE）（敏感度39.73%、特异度89.11%）和胃泌素前体释放肽（progastrin-releasing peptide, ProGRP）（敏感度51.48%、特异度94.89%）（表 1）。相同指标在国外相关文献中的统计结果见表 2。

**1:** 单项血清肿瘤标志物检测（国内） Detection of the single tumor marker in Serum (domestic)

Biomarker	Diagnosis	Reference	Patients	Controls	Sensitivity	Specificity	Accuracy
CEA	SCLC, NSCLC, adenoCA	52	6 754	3 942	47.50%	92.34%	64.03%
CA125	SCLC, NSCLC, adenoCA	42	3 082	1 186	50.11%	80.19%	58.47%
CYFRA21-1	NSCLC, SCC	53	4 142	2 734	57.00%	90.16%	70.19%
TPA	NSCLC, SCC	13	1 774	700	50.93%	88.41%	61.54%
SCCAg	NSCLC, SCC	8	814	510	49.00%	91.07%	65.20%
NSE	SCLC	47	3 857	2 914	39.73%	89.11%	60.98%
ProGRP	SCLC	7	503	473	51.48%	94.89%	72.52%
CA19-9	SCLC, NSCLC	19	2 404	2 075	44.00%	93.94%	67.13%
CA153	SCLC, NSCLC	26	2 598	3 110	39.66%	94.11%	69.33%
Ferrtin	SCLC, NSCLC	5	749	415	27.90%	86.27%	48.71%
GSF	SCLC, NSCLC	0	0	0	0	0	0
M-CSF	SCLC, NSCLC	0	0	0	0	0	0
DKK1	SCLC, NSCLC	1	592	192	69.50%	92.20%	85.70%
NSCLC: non-small cell lung cancer; CC: squamous cell carcinoma; adenoCA: adenocarcinoma; SCLC: small cell lung cancer.							

**2:** 单项血清肿瘤标志物检测（国际） Detection of the single tumor marker in serum (international)

Biomarker	Diagnosis	Reference	Patients	Controls	Sensitivity	Specificity	Accuracy
CEA	SCLC, NSCLC, adenoCA	43	5 425	2 058	33.23%	89.56%	48.72%
CA125	SCLC, NSCLC, adenoCA	9	1 907	382	28.51%	88.67%	38.55%
CYFRA21-1	NSCLC, SCC	51	9 737	6 301	46.96%	91.37%	64.41%
TPA	NSCLC, SCC	18	2 242	1 405	51.16%	86.90%	64.93%
SCCAg	NSCLC, SCC	14	2 029	955	29.71%	96.93%	51.22%
NSE	SCLC	23	2 641	1 593	43.31%	90.76%	61.16%
ProGRP	SCLC	6	883	722	57.08%	91.69%	72.64%
CA19-9	SCLC, NSCL	2	93	72	72.04%	84.72%	77.11%
CA153	SCLC, NSCL	2	76	60	68.42%	83.33%	75.00%
Ferrtin	SCLC, NSCL	4	306	102	48.69%	68.62%	53.68%
GSF	SCLC, NSCL	2	112	68	66.07%	67.85%	72.61%
M-CSF	SCLC, NSCL	2	112	68	56.25%	39.70%	50.00%
DKK1	SCLC, NSCL	0	0	0	0	0	0

### 2.2 联合指标统计结果

共统计国内外83篇联合检测的文献，肿瘤血清5 607例，对照血清4 077例。研究多以CEA和CA125联合其它指标检测肺癌，小细胞肺癌多联合NSE和ProGRP，尤以NSE+ProGRP对小细胞肺癌的诊断价值最大，敏感度和特异度分别为88.90%和72.82%。鳞癌多联合CYFRA21-1、SCCAg，TSGF+SCCAg+CYFRA21-1对鳞癌诊断的敏感度和特异度分别为95.3%和74.2%；腺癌多联合CA153、CA19-9等，CA153+Ferrtin+CEA对肺癌诊断的敏感度和特异度分别为91.90%和44.00%。联合指标的检测的临床运用价值及其特异度、敏感度、准确率见表 3。

**3:** 血清肿瘤标志物联合检测的诊断价值 The diagnostic significance of combined detection of tumor markers in serum

Tumor markers combination	Diagnosis	Groups		Sensitivity	Specificity	Accuracy
		Case	Control			
NSE+ProGRP	SCLC	338	244	88.90%	72.82%	82.16%
NSE+CYFRA21-1	NSCLC, SCC, SCLC	372	212	72.90%	90.30%	79.22%
CEA+NSE	SCLC, NSCLC, adenoCA	271	156	71.69%	93.58%	79.69%
TPS+NSE+CEA	SCLC, NSCLC, adenoCA	300	284	90.50%	84.20%	80.43%
CEA+CYFRA21-1	SCLC, NSCLC, adenoCA, SCC	213	172	80.19%	93.57%	86.17%
CEA+NSE+CYFRA21-1	SCLC, NSCLC, adenoCA, SCC	1 043	886	87.20%	86.30%	86.79%
CA125+CA50+CEA	SCLC, NSCLC, adenoCA	38	58	97.37%	78.57%	89.39%
CEA+CA125+CA19-9+CA153	SCLC, NSCLC, adenoCA	209	245	91.39%	88.45%	89.81%
CA125+CA19-9+CA153	SCLC, NSCLC, adenoCA	364	354	96.17%	72.96%	84.73%
CEA+CA125+CA19-9	SCLC, NSCLC, adenoCA	279	215	80.64%	84.45%	82.30%
CYFRA21-1+CA125+CEA	SCLC, NSCLC, adenoCA	188	73	85.46%	59.87%	78.30%
CEA+SCCAg+NSE	SCLC, NSCLC, adenoCA, SCC	253	223	85.80	94.00%	89.64%
CYFRA21-1+CA50+CEA	SCLC, NSCLC, adenoCA	131	29	79.10%	77.30%	78.40%
CYFRA21-1+CEA+NSE+CA125	SCLC, NSCLC, adenoCA, SCC	207	68	90.33%	97.50%	92.10%
CYFRA21-1+CA125+NSE	SCLC, NSCLC, adenoCA, SCC	111	80	88.55%	84.10%	86.69%
CA125+CEA+NSE	SCLC, NSCLC, adenoCA	109	90	73.10%	82.28%	77.25%
CEA+CA19-9+SCCAg	SCLC, NSCLC, adenoCA, SCC	78	45	70.50%	86.70%	74.40%
TPS+CYFRA21-1+NSE	NSCLC, SCC, SCLC	253	223	88.50%	90.00%	89.20%
CA153+ Ferrtin +CEA	SCLC, NSCLC, adenoCA	99	25	91.90%	44.00%	82.19%
NSE+CEA+CYFRA21-1+CA19-9	SCLC, NSCLC, adenoCA	95	48	88.42%	22.92%	66.43%
TSGF+SCCAg+CYFRA21-1	NSCLC, SCC	64	155	95.30%	74.20%	80.37%

## 3 讨论

肺癌血清肿瘤标志物检测意义如下：①肿瘤标志物的升高可先于顽固性咳嗽、痰中带血、癌性胸水等临床症状的出现，因此可通过检测血清异常增高的肿瘤标志物以确定肿瘤疾病的发生，实现早期诊断；②通过认识增高的血清肿瘤标志物种类以初步判断疾病的分期；如：研究^[4]^证明术前血清CEA水平可以作为肿瘤疾病TNM分期的参考标准；血清CYFR A21-1、TPA、CA125的异常增高与肿瘤疾病的分期明显相关^[5]^；③治疗中后期血清肿瘤标志物检测在评价治疗效果的同时有助于系统地改善治疗方案；如：研究^[6]^表明血清NSE水平虽然和肺癌的分期没有相关性，但可以作为对化疗是否敏感的参考指标，同时，对治疗后血清NSE水平的监测有助于对患者预后的判断^[7]^；针对治疗后患者血清ProGRP、NSE、CYFR A21-1和CEA水平的检测有助于判断小细胞肺癌患者是否对Ⅰ期化疗敏感从而考虑是否调整治疗方案^[8]^；④治疗后通过随访监测血清肿瘤标志物水平以及时预防肿瘤疾病的复发。

本*meta*分析表明：腺癌敏感标志物为CEA；鳞癌敏感标志物为CYFRA21-1、TPA和SCCAg；小细胞肺癌敏感标志物DKK1、NSE和ProGRP。所取得的结果与传统认为的不同肿瘤标志物在不同肺癌类型中的表现特点基本一致。我们也发现了血清肿瘤标志物在非肿瘤性疾病中的报道，如：Racil等^[9]^研究表明，NSE在肺结核患者血清中相对于正常人群有特异性增高，并可作为肺结核病的辅助诊断方法；ProGRP在肾衰竭患者血清中含量明显增高，需与小细胞肺癌引起的增高相鉴别，这些研究表明，肿瘤标志物在良性疾病中的应用价值也值得人们关注。

我们对23种不同联合检测进行统计，利用并联试验原理诊断肺癌，随着抗原种类的增多，诊断的灵敏度随之增加，敏感度均在70%以上，除了CA153+Ferrtin+CEA特异度低于45%以外，其余检测方法特异度皆在58%以上，所有检测指标的准确率均在70%以上，如：国外报道NSE单独检测肺癌的敏感度为43%左右，ProGRP单独检测的敏感度为57%左右，而二者联合检测，可将敏感度提高至85%以上，且特异度达到70%以上；国外报道CEA单独检测肺癌的敏感度为33.23%，特异度为89.56%，准确率为48.72%，而和CYFR A21-1联合用于肺癌检测时，敏感度增至80.19%，特异度增至93.57%，准确率为86.17%。同时，我们不断关注包括DDK1等新发现的肺癌血清肿瘤标志物，研究^[10]^表明，DKK1在小细胞肺癌的诊断中，敏感性高于NSE检测；DKK1+CYFRA21-1及DKK1+NSE对NSCLC、SCLC检测的敏感度可达80%-90%血清肿瘤标志物联合检测克服了单指标非特异性表达引起的假阳性结果，克服了检测指标在敏感度和特异度上不能兼顾的缺点，不仅在针对肿瘤治疗反应的监测、防止肿瘤的复发方面有着重要价值，同时在肿瘤的二级预防中也有着重要作用^[11]^，我们应该在合理选择肿瘤标志物的前提下，最大限度的确保诊断的准确性，这也是血清肿瘤标志物检测方法发展的必然趋势。

我们统计了712例13项肺癌相关血清肿瘤标志物，覆盖1994年1月-2009年9月所有相关文献，个别研究采用了小样本，总的样本量小于100或处于100-300的研究导致在评价指标表现特点方面不够精确，而有19项研究总的样本量超过了300例，这些结果可信度相对较高。在良性病变和对照组的选择上，病例选自于有典型症状患者，如选择那些有肺部炎性疾病的患者或者来自于同一所医院的有相似特征体检人群的研究结果可比性较高。少数肿瘤标志物，如：DKK1、集落刺激因子（colony stimulating factor, GSF）等，国内外报道的文献较少，仅代表少数研究的观点，这些检测指标值得我们继续关注。

本文统计分析的是标准化、有可重复性和质量可信的研究，部分有价值的报道因初始数据不全被排除，但仍不可避免存在以下不足：①在很多列举的研究中，研究人群的特征没有具体统计，且肿瘤分期对血清肿瘤标志物的影响没有单独阐明；②因为每篇报道使用的指标临界值有一定差异，故阳性/阴性病例选取上有一定偏倚；③这篇文章的观点仅仅代表了对于一些研究的总结，因为不同研究之间本身存在的异质性，以及实验材料和方法本身对敏感性的影响，所以根源偏倚在本文没有完全取舍。个别研究报道了意外的高表达，比如：联合检测CA125、CA50及CEA，其敏感度达97.37%，特异度达78.57%，但讨论的肿瘤血清样本仅38例，因此其结果的可信度值得商榷。部分针对同一指标的不同研究选择对象和样本没有分别使用独立研究和分子标记物确认和定量，所以这些实验方法可能会产生混杂和假阳性的结果。

传统的影像学检测不能提供直接的证据，痰或胸水细胞学检查存在较大的波动性，纤维支气管镜活检推广范性较局限，而血清肿瘤标志物检测同时具备取材便捷、结果可信度高等优点，有学者^[12]^提出将肿瘤标志物检测作为一项常规的体检项目开展。目前，联合检测指标尚缺乏标准化的检测方案，而建立合理的、经济的、快速的联合检测方案方法，才能真正实现肿瘤的早期诊断。在最近几年，关于肺癌肿瘤标志物的研究已经取得很大发展，更多人将注意力转到研发新的肿瘤标志物检测手段，以增加检测的敏感度和特异度，针对有伴发症的患者，如：血痰、胸水等患者，可进行多方面取材检测，如：有研究^[13]^表明联合ProGRP、NSE、CY- FRA2121、CEA对胸腔积液进行肿瘤标志物检测有助于肺癌的鉴别诊断和组织学分型；Fuchs等^[14]^研究发现，肺癌患者呼出的醛类气体可提示肿瘤的发生及其生长情况。在最近几年，报道了很多研发和评估针对肺癌血清肿瘤标志物检测新的方法，近期的报告^[15]^显示，一些检测方法有能力改善当前的检测现状，如纳米分子技术应用于肿瘤标志物的检测，在有效提高检测敏感性的同时具有快速、简便等优势。伴随中国人口老龄化进程的加速，对肺癌的检测作为一项基础的、广泛的检查，其适用性、推广性也应该考虑在其中，我们应该将更深层次研究放在疾病的早期阶段，在建立合理检测方案的同时把肿瘤标志物检测作为一项常规的针对于高危人群的肿瘤筛查方法，才能真正实现肺癌的早期发现、早期治疗，有效提高患者的生存率。
